# Cysteine Proteases: Modes of Activation and Future Prospects as Pharmacological Targets

**DOI:** 10.3389/fphar.2016.00107

**Published:** 2016-04-25

**Authors:** Sonia Verma, Rajnikant Dixit, Kailash C. Pandey

**Affiliations:** ^1^Host-Parasite Interaction Biology Group, National Institute of Malaria Research, Indian Council of Medical ResearchNew Delhi, India; ^2^Department of Biochemistry, National Institute for Research in Environmental Health, Indian Council of Medical ResearchBhopal, India

**Keywords:** auto-catalysis, *trans*-activation, pH sensor, prodomain, zymogen, protein–protein interaction

## Abstract

Proteolytic enzymes are crucial for a variety of biological processes in organisms ranging from lower (virus, bacteria, and parasite) to the higher organisms (mammals). Proteases cleave proteins into smaller fragments by catalyzing peptide bonds hydrolysis. Proteases are classified according to their catalytic site, and distributed into four major classes: cysteine proteases, serine proteases, aspartic proteases, and metalloproteases. This review will cover only cysteine proteases, papain family enzymes which are involved in multiple functions such as extracellular matrix turnover, antigen presentation, processing events, digestion, immune invasion, hemoglobin hydrolysis, parasite invasion, parasite egress, and processing surface proteins. Therefore, they are promising drug targets for various diseases. For preventing unwanted digestion, cysteine proteases are synthesized as zymogens, and contain a prodomain (regulatory) and a mature domain (catalytic). The prodomain acts as an endogenous inhibitor of the mature enzyme. For activation of the mature enzyme, removal of the prodomain is necessary and achieved by different modes. The pro-mature domain interaction can be categorized as protein–protein interactions (PPIs) and may be targeted in a range of diseases. Cysteine protease inhibitors are available that can block the active site but no such inhibitor available yet that can be targeted to block the pro-mature domain interactions and prevent it activation. This review specifically highlights the modes of activation (processing) of papain family enzymes, which involve auto-activation, *trans*-activation and also clarifies the future aspects of targeting PPIs to prevent the activation of cysteine proteases.

## Introduction

Cysteine proteases are present in all living organisms. Besides their fundamental functions of catabolism and protein processing, cysteine proteases perform diverse functions (Chapman et al., 1997; Vasiljeva et al., 2007). Cysteine proteases of parasites play key role in hemoglobin hydrolysis, blood cell invasion, egress, surface proteins processing (Lecaille et al., 2002; Sajid and McKerrow, 2002; Sijwali and Rosenthal, 2004). In 1937, papain was the first cysteine protease isolated and characterized from *Carica papaya* (Walsh, 2014). Papain and cathepsins belong to the most abundant family of the cysteine proteases. In mammals, a main group of cysteine proteases is known as lysosomal cathepsins (McGrath, 1999). The name cathepsin, is derived from the Greek kathepsein (to digest; Willstätter and Bamann, 1929). Bioinformatics analysis reveals that human genome encodes 11 cysteine cathepsins, i.e., the cathepsins B, C, F, H, K, L, O, S, V, X, and W, existing at the sequence level (Rossi et al., 2004). Cathepsins and other cysteine proteases from parasites as well as viruses may become good targets for major diseases such as arthritis, osteoporosis, AIDS, immune-related diseases, atherosclerosis, cancer, and for a wide variety of parasitic diseases such as malaria, amebiasis, chagas disease, leishmaniasis, or African sleeping sickness (Petrov et al., 2000; Lecaille et al., 2002; Gills et al., 2007; Salminen-Mankonen et al., 2007; Hirai et al., 2013).

In parasitic disease like malaria, cysteine proteases (falcipains) of *Plasmodium falciparum* specifically involve in hemoglobin degradation, parasite egress, processing surface proteins, therefore, function as a promising new drug targets (Francis et al., 1997; Rosenthal et al., 2002). *P. falciparum* expresses four papain-like cysteine proteases named as falcipain-1, 2, 2′ and 3. Falcipain-2 and -3 are the major cysteine proteases of *P. falciparum* involved in hemoglobin hydrolysis (Pandey et al., 2004, 2005; Sijwali and Rosenthal, 2004; Sijwali et al., 2006).

For preventing unwanted protein degradation, like other proteolytic enzymes (serine, aspartic, and metalloproteases), cysteine proteases are also synthesized as inactive precursors (or zymogens). Cysteine protease zymogens contain a prodomain that block access of substrate to the active site (Coulombe et al., 1996). Besides acting as an endogenous inhibitor (Pandey et al., 2004, 2009), prodomain may have additional roles in protein folding and or intracellular sorting (Tao et al., 1994; Cuozzo et al., 1995; Pandey et al., 2004). Activation of an enzyme from its zymogen generally takes place within a subcellular compartment or the extracellular environment, in which the particular enzyme performs its biological function. Zymogen conversion may be accomplished by accessory molecules (e.g., trypsinogen convert into trypsin in presence of ca^2+^), by an auto catalytic process with requirement of a significant drop in pH and by other enzymes as in *trans*-activation (Kay and Kassell, 1971; Mason and Massey, 1992). This review highlights the different modes of activation in cysteine proteases and their future aspects.

### Cysteine Proteases

**FIGURE 1:**
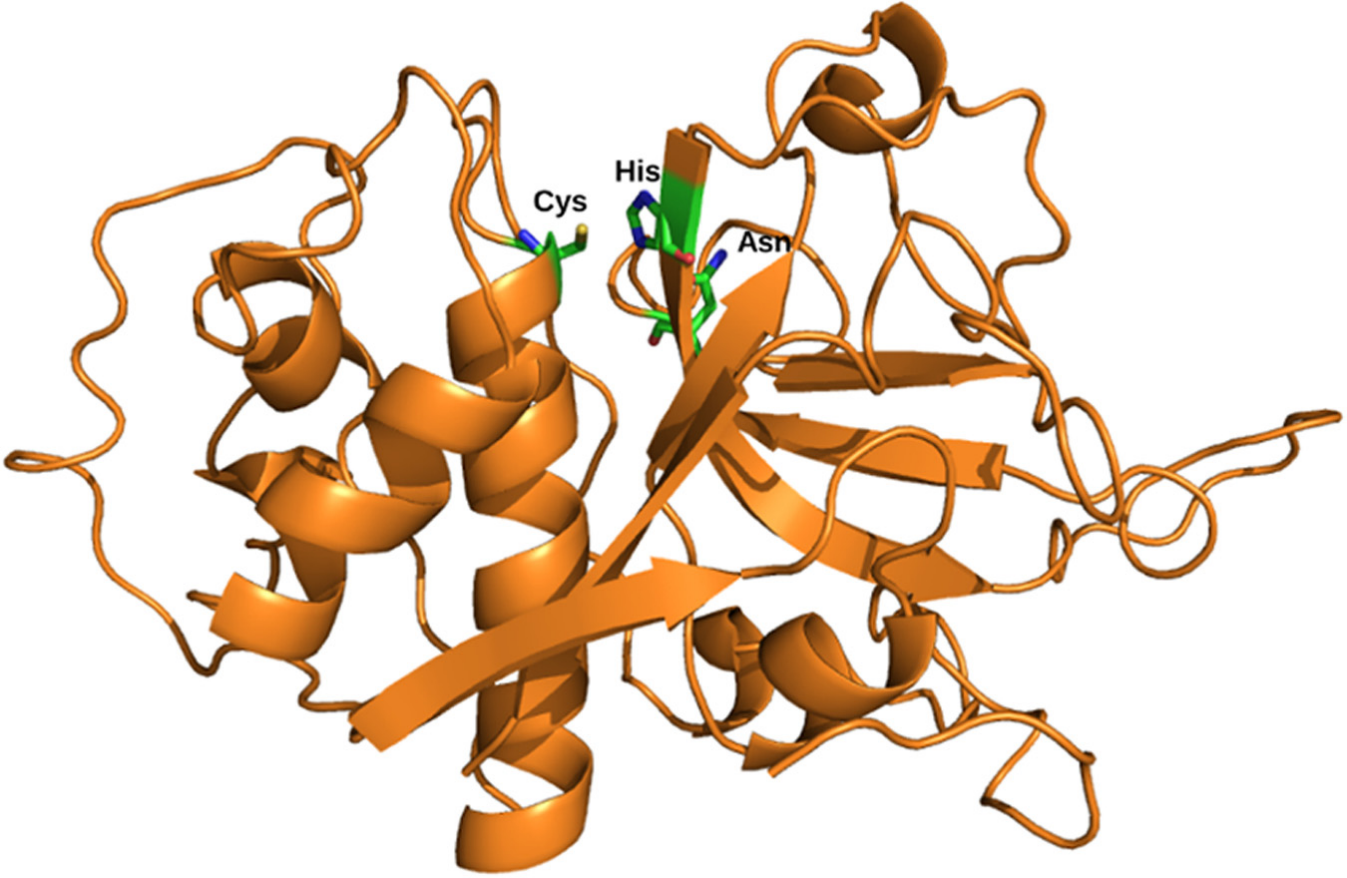
**Structure of the mature domain.** Structural representation of cysteine protease with the mature domain (*Orange*) showing active site residues; Cys, His, and Asn (*stick*); PBD ID: 3PNR.

**FIGURE 2:**
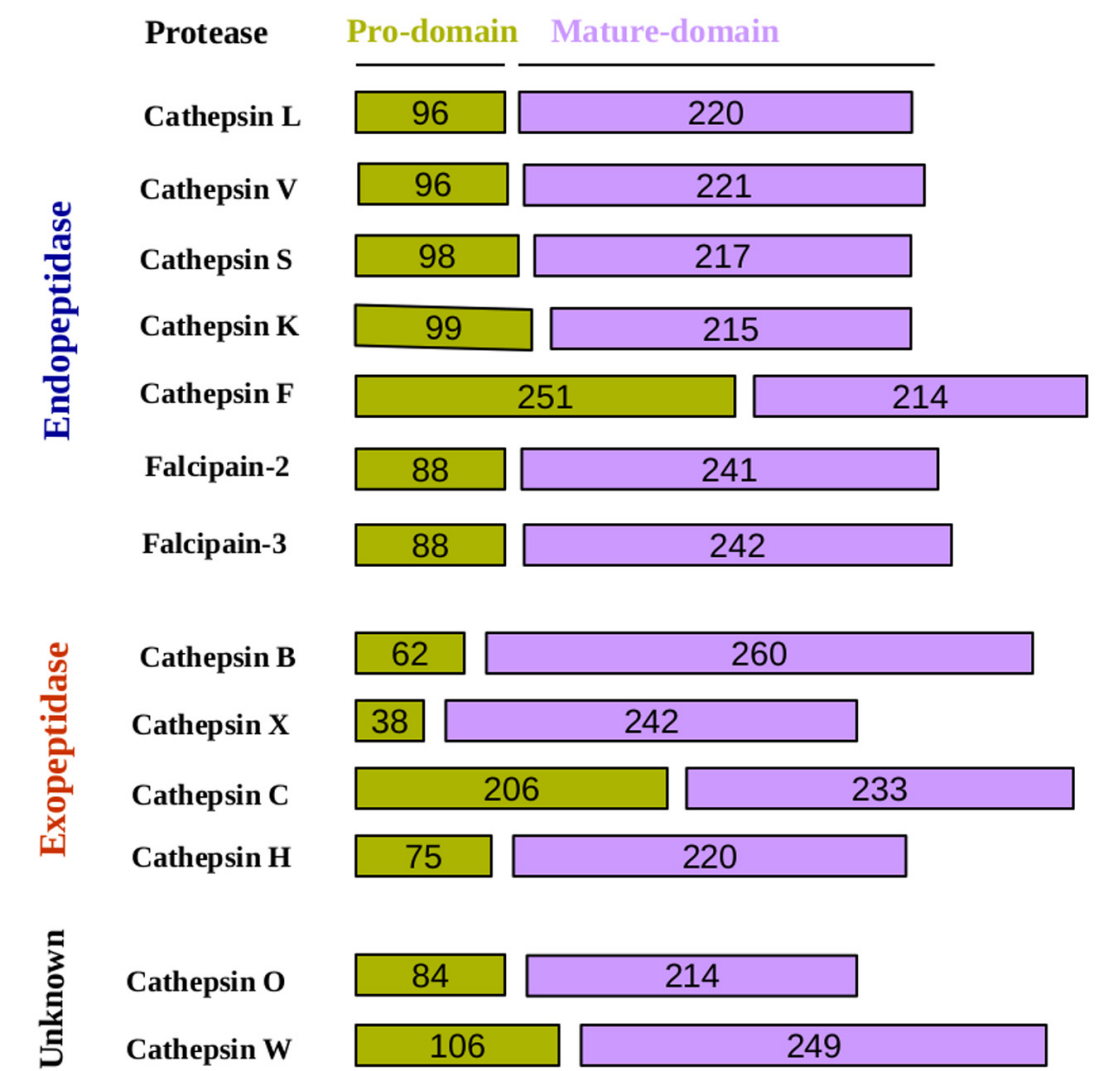
**Schematic representation of cysteine proteases pro and mature domain.** In this schematic representation, cysteine proteases have classified according to their peptidase property and depicted length of the domains.

Cysteine proteases contain a Cys–His–Asn triad at the active site. A histidine residue, presents in the active site act as proton donor and enhance the nucleophilicity of the cysteine residue (**Figure 1**). Nucleophilic cysteine residue attacks to the carbon of the reactive peptide bond, producing the first tetrahedral thioester intermediate in the reaction with release of an amine or amino terminus fragment of the substrate (Coulombe et al., 1996). This intermediate is stabilized by hydrogen bonding between the substrate oxyanion and a highly conserved glutamine residue. Subsequently, the thioester bond is hydrolyzed to produce a carboxylic acid moiety from the remaining substrate fragment. Based upon a sequence analysis of papain-like cysteine protease family was divided into two distinct subfamilies, cathepsin-L-like and cathepsin-B-like proteases, which can be distinguished by the structure of the prodomain and the mature domain (Coulombe et al., 1996; Turk et al., 1996). A third ‘F-like’ group was also proposed based on phylogenetic analyses showing that cathepsins F and W prodomains share a specific sequence pattern, ERFNAQ (Martinez and Diaz, 2008). The main difference between the sub-families exists in the sequence of the prodomains and their length (Dieter Bromme, 2011) (**Figure 2**). The prodomain of the cathepsin L subfamily (cathepsins L, V, K, S, W, F, and H) contain a prodomain of about 100 residues, with two conserved motifs: a highly conserved ERFNIN and GNFD motifs. An ERFNIN motif is lacking in cathepsin B, C, O, and X. Cathepsin B has a characteristic feature with ‘occluding loop’ which provide carboxydipeptidase activity (Turk et al., 1996). Falcipains, malarial cysteine proteases belongs to cathepsin L-like subfamily. Falcipains, malarial cysteine proteases have some unusual features, including large prodomains, predicted membrane-spanning sequences within the prodomains (Rosenthal, 2011).

### Zymogen Structure and Mechanism of Inhibition

The mature enzymes or active enzymes of the family share a well-known fold of papain-like which consists of two domains: N-terminal helical domain and a C-terminal β-sheet domain. Catalytic Cys, His, and Asn are contributed by these two domains. These residues are located in the substrate binding cleft (**Figure 1**).

**FIGURE 3:**
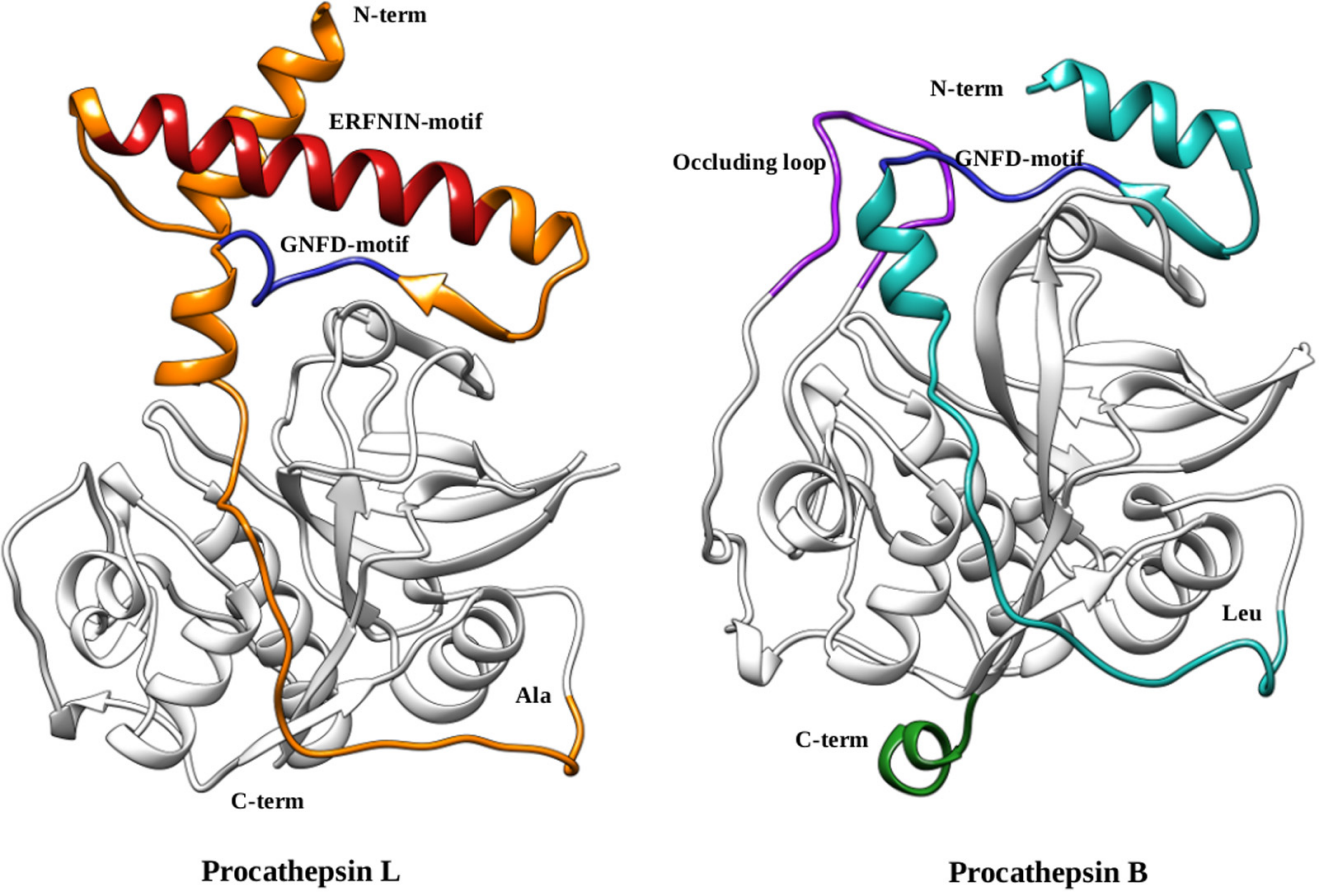
**Comparison of procathepsin L and B.** Structural representation of the procathepsin L (*orange*) (PDB ID: 1CS8) ERFNIN (*red*) and GNFD (*blue*) motifs are shown. The prodomain of cathepsin B (*cyan*) (PDB ID: 1MIR) with GNFD-motif (*blue*), occluding loop (*purple*), and long carboxy terminus (*green*) are mentioned in the structure.

The cathepsins B and L are well characterized papain-like enzymes, synthesized as zymogens (Coulombe et al., 1996; Turk et al., 1996). The prodomain of cathepsin L (96 amino acids) has an N-terminal globular domain that consist of three α-helices and their connecting loops, an extended structure followed by globular domain which traverse the substrate binding cleft in a reverse orientation (N→C-terminal) with respect to substrate that are cleaved and prevent access to active site. The prodomain and the mature domain interact via many residues (Phe^56^p, Phe^63^p, phe^71^p, Tyr^146^, and Tyr^151^; p stands for prodomain) lying at their interface (Coulombe et al., 1996). These residues are crucial for the activation of the mature enzyme. In falcipain-2, a mutation at prodomain residue Phe^60^p (equivalent to Phe^56^p of prodomain of cathepsin L) has shown inability to undergo auto-activation at its optimum pH (5.5) (Sundararaj et al., 2012). A conserved Gly^77^p residue helps in bringing the prodomain deep inside the catalytic cleft, in such a way that an oxyanion hole forms a hydrogen bond to Asn^76^p, and indirectly prevent access to catalytic cysteine for the catalysis. The prodomain of cathepsins B has similar fold as procathepsin L, but have some differences such as deletion of 30-residues from the N-terminal makes the prodomain short as compared to procathepsin L, orientation of the helices α2p and α3p are different due to presence of an ‘occluding loop’ a characteristic feature of the cathepsins B subfamily and absence of conserved ERFNIN motif (Turk et al., 1996). Due to presence of an occluding loop, cathepsins B subfamily is able to cleave dipeptide units from the carboxyl-terminal of the substrate (**Figure 3**).

Although there are differences but both the classes of enzymes have conserved mode of inhibition and share some common features such as interactions between the loop and the conserved hydrophobic residues in the prodomain and a conserved Gly residue near to the catalytic Cys residue. A conserved Gly in the hydrophobic pocket facilitate the C-terminus of the prodomain to push deep into the catalytic cleft and inhibit the catalytic site to perform catalysis. The prodomains exhibit a limited selectivity of inhibition against their related enzymes (Groves et al., 1998). First evidence for prodomains acting as selective inhibitors of cathepsin was seen when recombinant cathepsin L prodomain inhibited cathepsin L with lower Ki (0.088 nM) and cathepsin S with higher Ki (44.6 nM), whereas no inhibition of papain or cathepsin B was observed (Carmona et al., 1996). Besides acting as an endogenous inhibitor, prodomain are also involved in some other functions such as refolding and intracellular sorting of the enzyme. Like other papain-family cysteine proteases, prodomain of falcipain-2 is a potent inhibitor of the enzyme (Pandey et al., 2004). The C-terminal portion of the prodomain that includes “ERFNIN” and “GNFD” motifs, appear to mediate inhibition in many papain family proteases. The N-terminal portion of the prodomain mediates trafficking of the enzyme to the food vacuole, a site for the hemoglobin digestion (Subramanian et al., 2007, 2009). Many studies reported that whole prodomains are involved in correct folding of the enzyme while in falcipain-2 and falcipain-3, only N-terminal motif with 15 amino acid of the mature domain is required for refolding of enzyme (Pandey et al., 2004). The C-terminal part of the prodomain is sufficient for falcipains inhibition (Pandey et al., 2009; Pandey and Dixit, 2012).

## Activation Mechanisms

Once reaching to its specific compartment, processing of the enzyme starts, which include cleavage of the prodomain and activation of the mature enzyme (Hasilik et al., 2009). In processing of cysteine protease, pH change has great importance. In lysosomes or food vacuoles, enzymes get activated by controlled proteolysis which involves autocatalysis or *trans*-activation (Schroder et al., 2010). Based upon previous studies on procathepsin L, a pH-dependent conversion may start with the disruption of the conserved salt bridges (Asp^65^→Arg^21^ and Gln^70^→Arg^31^) within the prodomain (Vernet et al., 1995). Disruption of the salt bridges due to protonation of the carboxylate group at the lower pH could conceivably trigger the disruption of the hydrophobic core of the prodomain, leading to dissociation of the prodomain from the active site, and thereby initiating the process of auto catalytic processing (Vernet et al., 1995).

Due to presence of an occluding loop in procathepsin B, mature domain undergoes significant conformation changes during the processing. In contrast, procathepsin L-like papain-family proteases do not have occluding loop, therefore, the mature domain is not expected to undergo significant change during the processing (Coulombe et al., 1996; Cygler et al., 1996).

Further, structures of other procathepsins provided insight in understanding zymogen activation. The crystal structures of human and rat procathepsin B (Cygler et al., 1996; Turk et al., 1996), human procathepsin L (Coulombe et al., 1996), human procathepsin K (Sivaraman et al., 1999), and human procathepsin X (Sivaraman et al., 2000) showed that the prodomain folds on the surface of the enzyme in an extended conformation and runs through the active-site, in the opposite direction to the substrate, thereby blocking the access of the active site. In the structure of most proenzymes, salt bridges, hydrogen bonding, and hydrophobic interactions within the prodomain and between the pro-mature domains exist. The exception is the structure of cathepsin X, in which the prodomain binds covalently to the mature enzyme with a disulphide bridge between cysteine residue in the prodomain and active site cysteine, thus preventing any auto-activation (Sivaraman et al., 2000). However, it can be processed *in vitro* under reducing conditions by cathepsin L (Nagler and Menard, 1998).

Processing or zymogen activation of proteases may exhibit via different modes such as auto-activation and *trans*-activation or both (Nishimura et al., 1989; Menard et al., 1998). Auto-catalysis involves the cleavage of prodomain by catalytic site present inside the catalytic cleft of the enzyme under the influence of pH change (Menard et al., 1998). However, *trans*-activation involve cleavage by the another molecule of the same enzyme or some other proteases that cleave within the residues lying at the junction of the prodomain and the mature domain such as pepsin, aspartic cathepsin D, and legumain/asparaginyl endopeptidase (Nishimura et al., 1989; Menard et al., 1998). It is reported that some proteases have unusual kind of activation such as Cathepsin D (Laurent-Matha et al., 2006). This review further highlights different modes of processing of cysteine protease zymogens.

### Activation by Auto-processing

**FIGURE 4:**
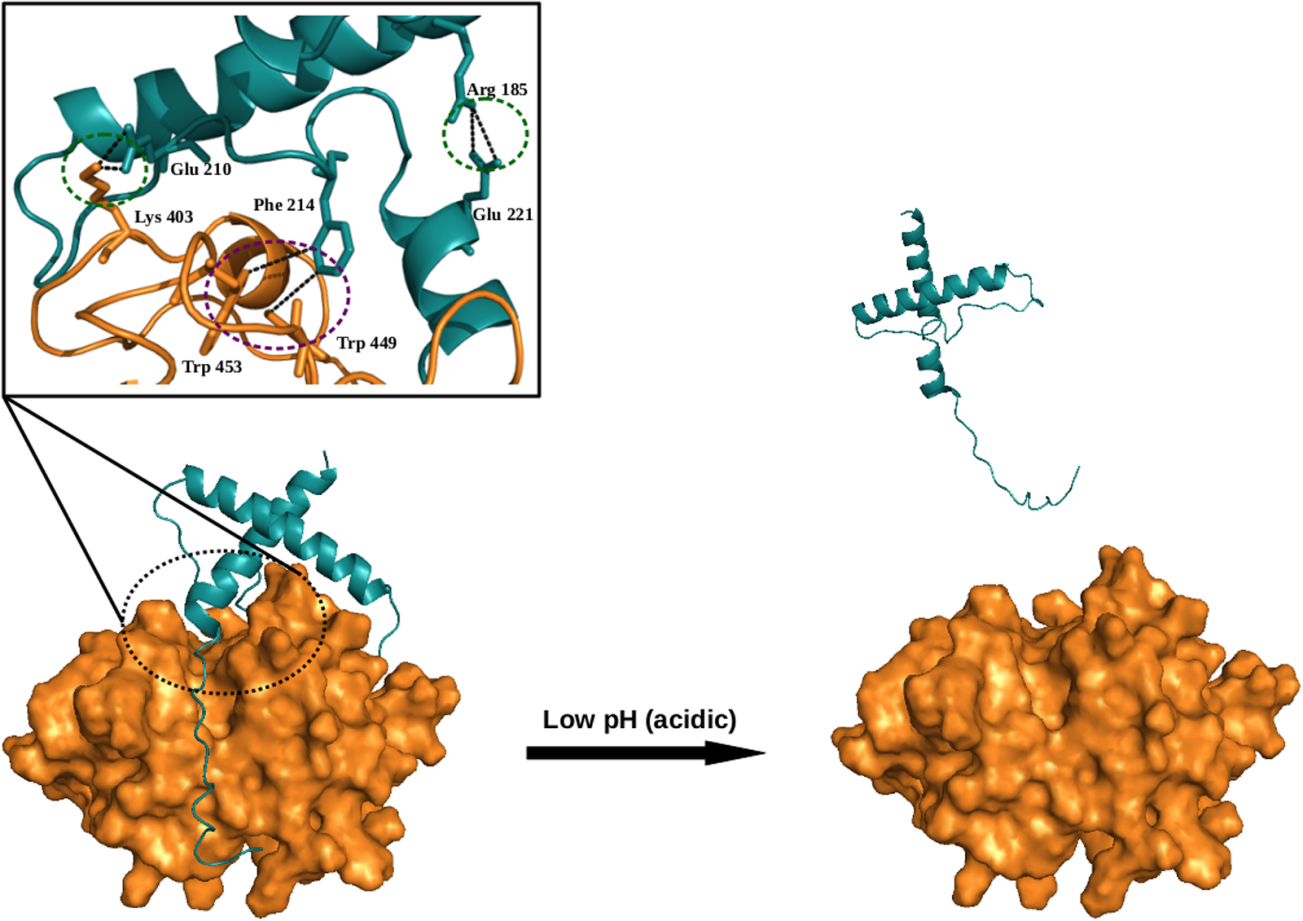
**Model (illustrative) for auto-activation of falcipain-2 (FP2), a malarial cysteine protease.** The prodomain of FP2 removed and enzyme get activated under the influence of low pH. Inset shows crucial salt bridges (*green-dotted circles*) and hydrophobic (*purple-dotted circle*) interactions are required for auto-activation. Model was generated via Modeler by taking known structures as templates (PDB ID; 3PNR and 1CS8). All figures are prepared using PyMOL.

Auto-catalysis is a common mode of activation of cysteine proteases itself from their zymogens under the influence of pH change (acidic pH). The pH has great importance in auto catalytic mode of activation. The pH change triggers the disruption of important interactions between the prodomain and the mature domain, thereby make accessible the cleavage site within the prodomain loop to the active site. An N-terminal prodomain get cleaved off from the whole enzyme by active site and enzyme get activated (**Figure 4**).

Early studies suggested that autocatalytic processing is a unimolecular process (Rowan et al., 1992; Mach et al., 1994) and, while others proposed inter-molecular and intra-molecular mechanisms (McQueney et al., 1997; Menard et al., 1998). However, this dilemma has been resolved that the auto-activation of cathepsins is a combination of a unimolecular and a bimolecular process (Rozman et al., 1999). For example, procathepsin B possesses a low catalytic activity that is not sufficient to trigger the auto catalytic activation. Low activity of cathepsin B is the result of the prodomain dissociation from the active-site cleft as the first step, which is a unimolecular step (Pungercar et al., 2009). In the next step, which is bimolecular, this catalytically active cathepsin B molecule processes and activates another procathepsin B molecule in one or more steps. The mature cathepsin B molecules generated in this way then initiate a chain reaction leading to a rapid activation of the remaining procathepsin B molecules. Cysteine proteases based on their cleavage property can be categorized into two groups; endopeptidase and exopeptidase (**Table 1**). Endopeptidases such as the cathepsins B, H, L, S, and K can be activated by autoactivation, whereas the true exopeptidases, such as the cathepsins C and X, needed endopeptidases, such as the cathepsins L and S (Dahl et al., 2001), for their activation (**Table 1**; **Figure 2**) (Dieter Bromme, 2011). Mutational studies of cathepsin K confirmed its mode of activation by autocatalysis. An active site Cys^139^ to Ser mutant of procathepsin K failed to undergo activation but could be processed fully when incubated with wild-type procathepsin K (McQueney et al., 1997).

**Table 1:** Types of cysteine proteases on the basis of cleavage property and their mode of activation.

Enzyme cleavage property	Enzyme (cysteine protease)	Activation mode
Endopeptidase	Cathepsin L, S, K, V, F, Falcipains	Auto-activation
Exopeptidase	Cathepsin C, X,	*Trans*-activation
Endo and Exopeptidase	Cathepsin B, H	Auto-activation


**FIGURE 5:**
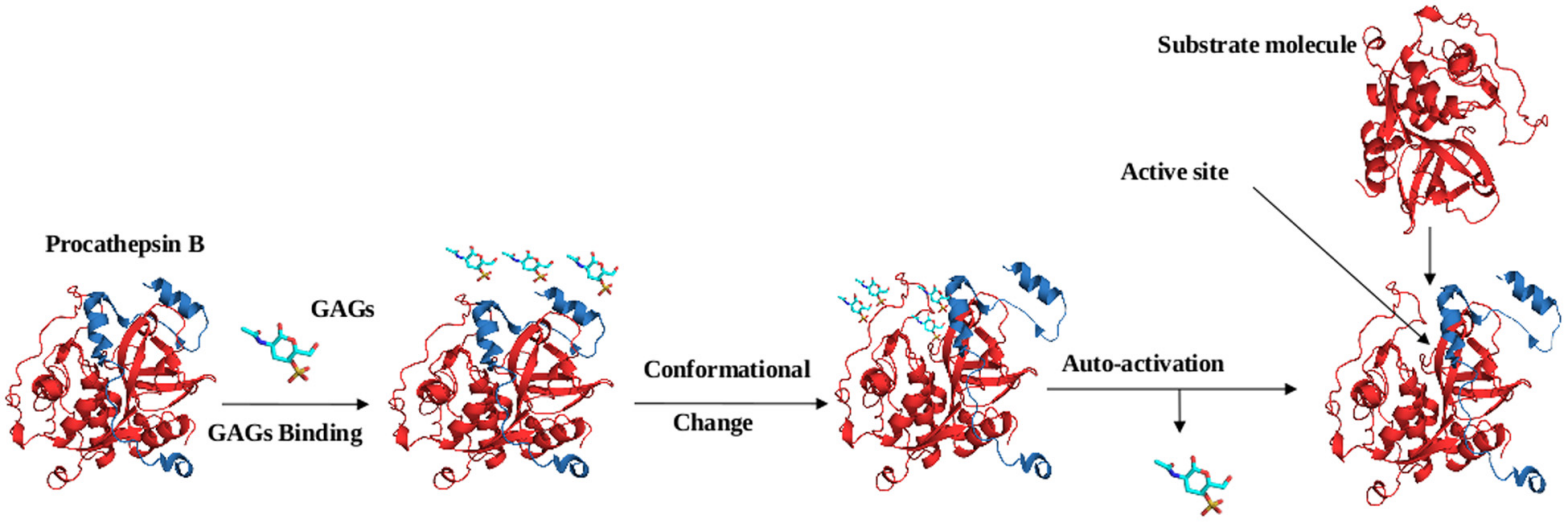
**Mechanism of procathepsin B auto-activation in the presence of glycosaminoglycans (GAGs).** GAG molecules bind to the occluding loop and the prodomain of cathepsin B molecule via electrostatic interactions. GAGs binding induce conformational change in procathepsin B (PDB ID: 1MIR), which unmasks the active site and enable the access of procathepsin B molecule.

The activation of cysteine proteases are modulated by the glycosaminoglycans (GAGs) such as sulfated GAGs, heparin, heparan sulfate, and chondroitin sulfates A, B, C, E, and other negatively charged polysaccharides, e.g., dextran sulfate. Various studies have suggested the involvement of GAGs in the *in vivo* processing of cathepsins (Caglic et al., 2007; Novinec et al., 2014). Similarly, GAGs accelerate the auto catalytic activation of cathepsin L and B, including at neutral pH. Studies reported that GAGs interact with cathepsin B via electrostatic interactions, being negatively charged, GAGs interact with the positively charged residues present in the occluding loop of the mature domain and the prodomain of the cathepsin B. GAGs binding induce conformation changes in the prodomain of cathepsin B, which unmask the active site for the catalysis of the other procathepsin B molecules (**Figure 5**) (Caglic et al., 2007). Further GAGs role in activation of cathepsins was confirmed by the auto-activation of procathepsin S at neutral pH (Vasiljeva et al., 2005). However, a recent finding with cathepsin S at high concentration of chondroitin-4-sulfate (C4S) exhibits a decelerating effect of GAGs on activation (Sage et al., 2013). Besides the activation of cysteine proteases, GAGs also have been found to affect both the activity and stability of the mature cysteine cathepsins (Almeida et al., 1999; Li et al., 2000). Most of the knowledge about the GAGs role in regulation of cathepsins came from the studies of papain. A hyperbolic kinetic profile of cathepsin K in presence of GAGs suggest their interactions outside the active site, may be via allosteric mechanisms (Li et al., 2008; Novinec et al., 2010).

Cysteine proteases play important role in life cycle of parasitic organism. Parasites also express cathepsin L and B like proteases. Falcipains, well characterized cathepsin L-like cysteine proteases from *P. falciparum*, synthesize as zymogens and get activated by auto activation in acidic environment (pH 5–5.5). Sundararaj et al. (2012), from our group revealed that prodomain-mature domain of falcipain-2 and falcipain-3 interacts via salt bridges and hydrophobic interactions. Mutagenesis study showed that two salt bridges (Arg^185^–Glu^221^, Glu^210^–Lys^403^) in falcipain-2, and one salt bridge (Arg^202^–Glu^238^) in falcipain-3, and hydrophobic interactions present both in falcipain-2 (Phe^214^–Trp^449^Trp ^453^), and falcipain-3 (Phe^231^–Trp^457^Trp ^461^) also play important roles in the activation of these enzymes (**Figure 4**). Mutants were unable to undergo autocatalysis, although, activation can further be achieved by *trans*-activation (Sundararaj et al., 2012).

Auto activation of cysteine proteases from other parasitic organisms have been proposed by many studies (Dalton et al., 1997; Stack et al., 2008). *In vitro* study about *Fasciola hapatica* procathepsin L1 (FhproCL1) demonstrated that auto activation can occur within wide pH range 4.5–7.3 (Lowther et al., 2009). Active site mutant of *F. hepatica* (FhproCL1Gly^25^) cannot undergo auto-catalytic processing (Collins et al., 2004). *Schistosoma mansoni*, a parasitic worm causing Schistosomiasis (also known as bilharzia) expresses papain-like cysteine proteases including cathepsins (B1, L1, L3, F, and C) and an aspartic protease (cathepsin D) in the parasite gut, function in an acidic pH environment (Gotz and Klinkert, 1993; Dalton and Brindley, 1996). A number of reports have shown that both parasite and mammalian cysteine proteases can be auto catalytically activated *in vitro* from zymogen to mature enzyme by reducing the pH of the solution (Lowther et al., 2009).

### Activation by *Trans*-processing

**FIGURE 6:**
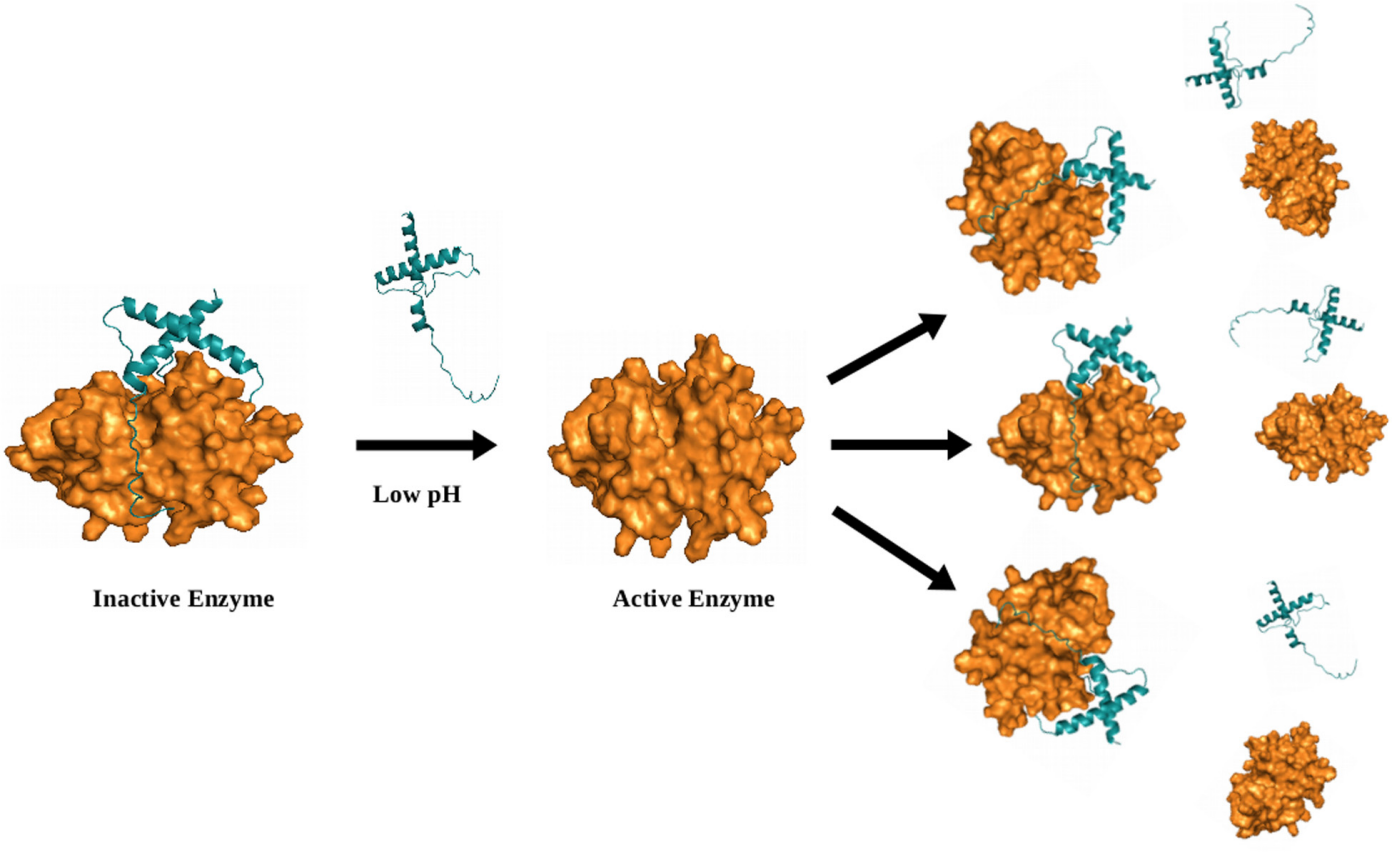
**Model (illustrative) for *trans*-activation by same enzyme molecule.** *Trans*-activation of the enzyme by other active molecule of same enzyme is shown the figure. Initially, single molecule of enzyme get activated under low pH, thereafter these active molecule further activate the other inactive enzymes and initiate a chain reaction. Model was generated via Modeler by taking known structures as templates (PDB ID; 3PNR and 1CS8).

**FIGURE 7:**
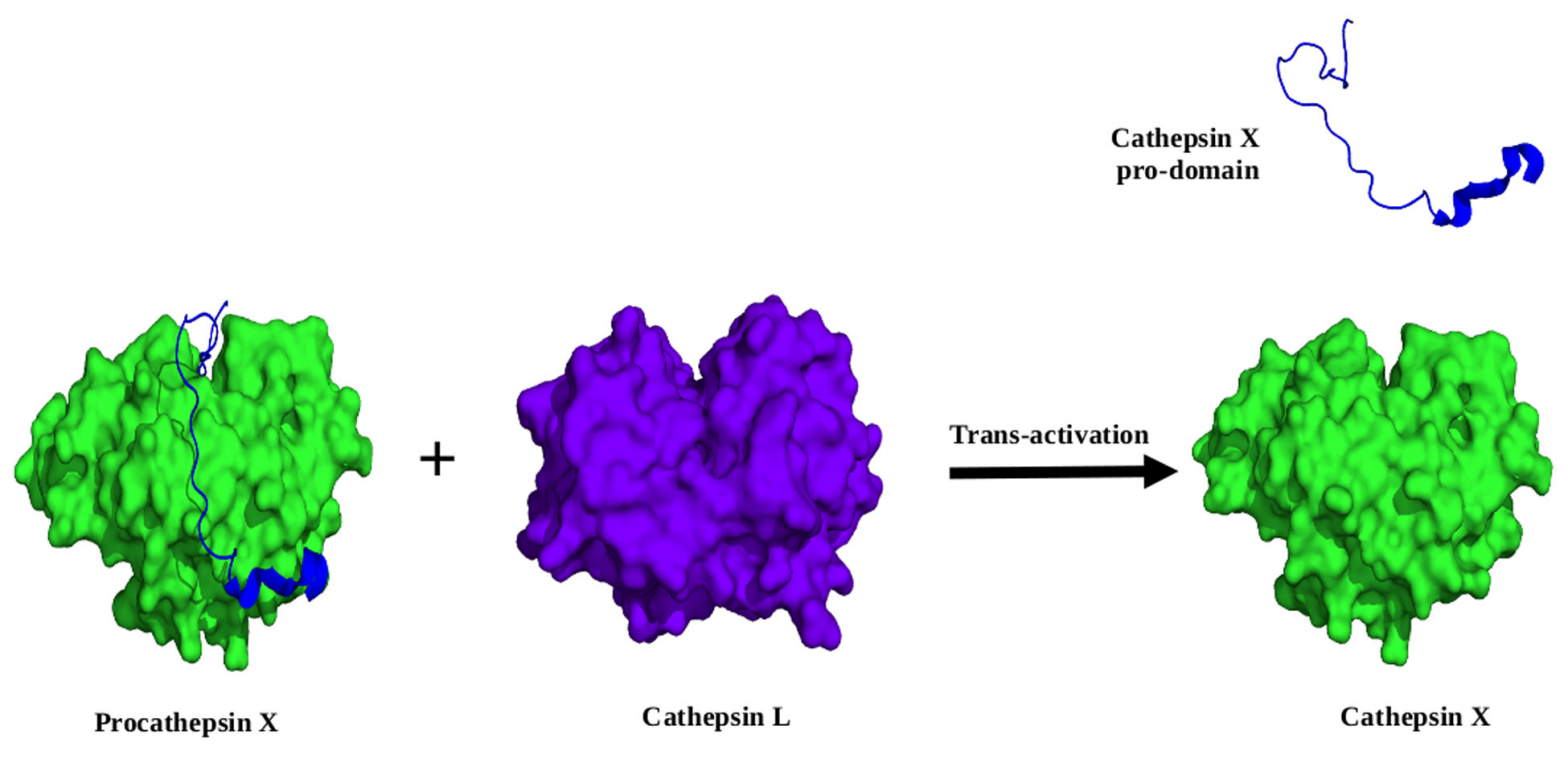
**Model (illustrative) of *trans*-activation by other enzyme molecule.** Procathepsin X (PDB ID: 1DEU) consists covalently linked prodomain (*blue*). Cathepsin L (*violet*) (PDB ID: 1CS8) *trans*-activate procathepsin X by cleaving the prodomain and releasing an active cathepsin X (*green*).

*Trans*-activation involve cleavage by another molecule of the same enzyme (**Figure 6**) or some other proteases that cleave within the residues lying at the junction of prodomain and mature domain such as pepsin, Cathepsin D, and legumain/asparaginyl endopeptidase (**Figure 7**) (Dalton and Brindley, 1996; Dahl et al., 2001). Cathepsin C and X require an endopeptidase such as cathepsins L or S for their activation. Moreover, cathepsin S prodomain was found to be rapidly degraded by cathepsin L. In contrast to most other cathepsins, cathepsin C was not capable of auto activation, even addition of the mature cathepsin C. This is consistent with procathepsin X, which is also an exopeptidase. Cathepsin X prodomain binds covalently to the mature enzyme with a disulphide bridge between cysteine residues in the prodomain and the active-site cysteine, thus preventing any auto-catalytic processing (Sivaraman et al., 2000). However, it can be processed *in vitro* under reducing conditions by cathepsin L and S (**Figure 7**) (Dahl et al., 2001). Falcipains zymogens can be *trans*-activated by their mature enzymes. In other parasites, cysteine proteases can be *trans*-activated by their mature enzymes and asparaginyl endopeptidase, a clan CD cysteine protease that cleaves C-terminal to asparginyl (Asn) residues (Dalton and Brindley, 1996). Pre-activated wild *F. hepatica* cathepsin L (FhproCL1) enzyme is able to *trans*-process (FhproCL1) zymogens at specific cleavage site Leu^12^–Ser^11↓^His^10^motif and thus increase rate of activation exponentially. Mutational study has shown that alteration of the motif to a Pro^12^–Ser^11↓^His^10^ prevents or slows down FhproCL1 activation (Stack et al., 2008). A study by Dalton et al. proposed that Schistosome asparaginyl endopeptidase (SmAE) is responsible for *trans*-processing of the cysteine proteases involved in the hemoglobin degradation in Schistosome (Dalton and Brindley, 1996; Sajid et al., 2003). Furthermore, *Fasciola hepatica* cathepsin B also have preserved asparaginyl endopeptidase-processing site at the pro-mature domain junction and SmAE was also shown to *trans*-process *F. hepatica* cathepsin B. Interestingly, the prodomain of *O. viverrini* cathepsin F (Ov-CF-1) lacks the conserved asparagine residues found in other homologues and could not be *trans*-processed by *Opisthorchis viverrini* asparaginyl endopeptidase. However, recent studies shown *O. viverrini* cathepsin B (Ov-CB-1) is capable of *trans*-activating cathepsin F (Ov-CF-1) at a specific site between the prodomain and the mature enzyme (Sripa et al., 2010).

### Activation by Uncommon Mode of Processing

Number of studies reported that both parasite and mammalian cysteine proteases can be auto activated *in vitr*o from zymogen to mature enzyme by reducing the pH of solution (Dalton et al., 2009), but some studies reported uncommon mode of activation which involves partial auto-activation followed by full activation by an enzyme.

The schistosome cathepsin B1 (SmCB1) is a unique in terms of activation, as the zymogen does not auto catalytically activate at low pH (Sajid et al., 2003; Dalton et al., 2009). Crystal structure of SmCB1 zymogen has solved and revealed another activation trigger which involved sulfate polysaccharides (Dalton and Dvorak, 2014). The prodomain of the SmCB1 contains a short and unique helix, α-3p which contains Schistosome asparaginyl endopeptidase (SmAE) cleavage site and a heparin-motif (LRRTRRP). A heparin-motif of the prodomain is involved in interaction with the mature enzyme and makes α-3p an ideal point of interaction for sulfated polysaccharides-like dextran sulfate. At low pH, SmCB1 zymogen auto catalytically produces an inactive intermediate clipping off 38 residues of the prodomain, but this does not fully expose the active site. Adding dextran sulfate to the reaction mixture facilitates the pinch off few more residues in the vicinity of substrate binding site, to produce an active intermediate. This finally, leads to processing of SmCB1 to the mature enzyme via *trans*-activation by SmAE. The binding of sulfated polysaccharides to an intact α-3p heparin-binding motif is required for complete processing of the SmCB1.

Cathepsin-D, an aspartic protease also synthesize as inactive enzyme. The activation of cathepsin-D, an intermediate mechanism had been proposed, i.e., a partial auto-activation followed by a final processing by other enzymes. Studies have shown that the processing of endocytosed procathepsin-D is also independent of its catalytic function and requires cysteine proteases for its activation (Laurent-Matha et al., 2006).

## Discussion

Proteases have been categorized into groups on the basis of the catalytic mechanism used during the hydrolytic process such as cysteine, serine, aspartate, and metalloproteases. For preventing unwanted degradation, cysteine proteases are synthesized as inactive enzymes and removal of the prodomains lead to the activation of the enzymes. Each class has their own mechanism of inhibition and activation. Main concept of inhibition is that prodomain sterically block the pre-formed active site and inhibit the access to the substrate (Khan and James, 1998). Generally zymogens get self-activated in low pH upon reaching to the specific compartments and it is reported that salt bridges plays important role in stabilization of pro-mature domain interactions at neutral pH. Upon reaching to specific compartment, prodomain are removed by autocatalysis or by other enzymes (*trans*-activation). Auto-activation is most economical mode of conversion since no involvement of other enzymes. However, *trans*-activation by other enzyme is required to enhance the conversion rate in critical conditions. For example, cathepsins F, a liver fluke (*O. viverrini*) enzyme, secreted as an inactive zymogen that auto-catalytically processes and activated to a mature enzyme at pH 4.5, but studies shows that it can also be activated via *trans*-processing by cathepsins B1 at pH 5.5, where it is unable to activate auto-catalytically. Both enzymes (cathepsin B1 and F1) work together to degrade the host tissue and contributing to develop liver-fluke-associated chalangiocarcinoma (Sripa et al., 2010).

*In vivo* studies reported, after removal, prodomains are hydrolyzed and ensure that the activation process is irreversible (Rozman et al., 1999; Turk et al., 2012). Although, *in vitro* study by Sijwali et al. (2002) showed that the prodomain of falcipain-2 is a potent, competitive, and reversible inhibitor of the active product. **Table 2** summarizes the mechanism of inhibition and activation of cysteine proteases.

**Table 2:** Cysteine protease with their inactivation and activation modes.

Enzyme class	Inactivation mechanism	Activation/Processing
Cysteine protease (Cathepsins and parasitic)	• Steric block of the active site by prodomain	• Generally via auto-activation but some are activated by *trans*-activation; e.g., cathepsin C and X
		• Disruption of salt bridges, hydrophobic interactions, e.g., falcipain-2 and falcipain-3
		• No conformational change in the mature domain during activation in cathepsin L-like cysteine proteases but cathepsin B-like undergo significant change due to occluding loop
		• Some proteases are involved in *trans*-activation, i.e., asparaginyl amino-peptidase


The pro-mature domain interactions are categorized in protein–protein interactions (PPIs). Presently, targeting PPIs is an attractive and promising area of research. A special interest in cysteine proteases as targets derives from the recognition that they are critical to the life cycle or pathogenicity of many parasites. This functional diversity is derived from their unique nucleophilicity, adaptability to different substrates, stability in different biological environments, and their regulations. Parasite derived cysteine proteases play key roles in hemoglobin hydrolysis, breakdown of RBC proteins, immunoevasion, enzyme activation, virulence, tissue, and cellular invasion as well as excystment, hatching, and molting (Sajid and McKerrow, 2002; Sijwali and Rosenthal, 2004). Previous study by Sundararaj et al. (2012) showed that the prodomain-mature domain interactions are essential for the auto-activation of the falcipains and their mutation impaired the enzyme ability to undergo activation by autocatalysis. Blocking of such interactions will cease the parasite growth due to inactivity of these proteases. Therefore, designing inhibitors against such interactions are important new targets.

## Future Aspects

In this review, we have discussed targeting the cysteine proteases before activation, which may prevent their involvement in various diseases. The possible ways are:

1. Targeting promature domain interactions which are important for the activation.

2. Targeting the residues involved in pH sensing and activation.

Pro-mature domain interactions can be categorized as PPIs. Earlier targeting PPIs were not in trend due to technological hurdles. Studies have demonstrated that small molecules can disrupt the large and complex protein interactions by interacting with interface residues, known as hot-spots (Mullard, 2012; Pandey, 2013). In a study of a herpes virus protease, researchers developed a small-inhibitor that target to block the interactions of two monomers, and prevent it forming the active dimer interface (Shahian et al., 2009). Recently, our group is also focusing on PPIs crucial for activation of enzyme in malarial cysteine proteases. Our earlier reports suggested that salt bridges and hydrophobic interactions between pro-mature domains were crucial for the activation of malarial cysteine proteases (Sundararaj et al., 2012), and disruption of these interactions lead to failure of the activation of enzymes. Therefore, it would be possible to control the growth of the parasites and other harmful organisms by blocking the processing of cysteine proteases. In our lab, we have screened some inhibitors, which are able to block the processing of falcipains and further detail characterization and kinetics of potential inhibitors are underway.

The pH has great importance in activation of zymogens of proteases in specific compartment (Schroder et al., 2010). It is very interesting, how pH triggers the activation? Inactive enzyme move through different compartments having varying pH gradient and get activated inside the specific compartment under the influence of pH change. Therefore, it is suggests that there are some sensors residues who can sense the pH change and trigger activation process (Feliciangeli et al., 2006). It is reported that folding and activation of furin, a calcium-dependent serine endoprotease, occurs through pH and compartment-specific auto-proteolytic steps. A conserved His^69^ of prodomain act as pH sensor that regulates the compartment-specific cleavages of the prodomain. Although, structural modeling combined with mathematical modeling and molecular dynamic simulations suggested that His^69^ does not contribute directly to the prodomain–enzyme interface. But, rather, triggers movement of a loop region in the prodomain that modulates access to the cleavage site and thus, allows the tight pH regulation of furin activation (Feliciangeli et al., 2006; Williamson et al., 2013). Like serine proteases, cysteine proteases also follow the similar mechanism to get activated auto-catalytically under the influence of compartment specific pH change. The cysteine proteases could have pH sensor residue like furin that sense pH change and trigger activation events. Taking this idea, we performed sequence alignment of the prodomain of the cysteine proteases among the organisms including malarial cysteine proteases and found a conserved His residue for further exploring the role as a pH sensor in processing of malarial cysteine proteases. Interestingly, our initial results support that notion that pH sensor also play important role in processing of falcipains. Therefore, disturbing the PPIs and designing inhibitor based on such interactions are futuristic approaches for preventing the pathogenic diseases, and they may have least sensitive to drug resistance.

## Author Contributions

SV and KP conceived and designed the review. SV, KP, and RD wrote the review paper.

## Conflict of Interest Statement

The authors declare that the research was conducted in the absence of any commercial or financial relationships that could be construed as a potential conflict of interest.
